# Ketogenic Metabolism in Neurodegenerative Diseases: Mechanisms of Action and Therapeutic Potential

**DOI:** 10.3390/metabo15080508

**Published:** 2025-07-31

**Authors:** Marta Pawłowska, Joanna Kruszka, Marta Porzych, Jakub Garbarek, Jarosław Nuszkiewicz

**Affiliations:** 1 Department of Medical Biology and Biochemistry, Faculty of Medicine, Ludwik Rydygier Collegium Medicum in Bydgoszcz, Nicolaus Copernicus University in Toruń, 24 Karłowicza St., 85-092 Bydgoszcz, Poland; 2 Student Research Club of Medical Biology and Biochemistry, Department of Medical Biology and Biochemistry, Faculty of Medicine, Ludwik Rydygier Collegium Medicum in Bydgoszcz, Nicolaus Copernicus University in Toruń, 24 Karłowicza St., 85-092 Bydgoszcz, Poland

**Keywords:** Alzheimer’s disease, amyotrophic lateral sclerosis, beta-hydroxybutyrate, cognitive decline, ketogenic diet, ketone bodies, mitochondrial dysfunction, neuroinflammation, Parkinson’s disease

## Abstract

Neurodegenerative diseases, including Alzheimer’s disease, Parkinson’s disease, and amyotrophic lateral sclerosis, are characterized by progressive neuronal loss and share key pathological features such as oxidative stress, mitochondrial dysfunction, and chronic neuroinflammation. Recent research has highlighted the potential of ketogenic metabolism, particularly the use of ketone bodies like β-hydroxybutyrate, as a therapeutic approach targeting these shared mechanisms. This review provides a comprehensive synthesis of current knowledge on the neuroprotective effects of ketogenic interventions, including both dietary strategies and exogenous ketone supplementation. We discuss how ketone bodies improve mitochondrial function, reduce reactive oxygen species, modulate inflammatory pathways, and influence neurotransmission and synaptic plasticity. Additionally, we examine experimental and clinical evidence supporting the application of ketogenic therapies in neurodegenerative diseases, highlighting disease-specific findings, benefits, and limitations. While preclinical data are robust and suggest meaningful therapeutic potential, clinical studies remain limited and heterogeneous, with challenges related to adherence, safety, and patient selection. The review also addresses the translational relevance of ketogenic strategies, considering their feasibility, combination with other therapies, and the need for personalized approaches based on genetic and metabolic profiles. By critically evaluating existing data, this article aims to clarify the mechanisms through which ketogenic metabolism may exert neuroprotective effects and to outline future directions for research and clinical application in the context of neurodegenerative disorders.

## 1. Introduction

### 1.1. Neurodegenerative Disorders: Shared Mechanisms

Major neurodegenerative diseases, such as Alzheimer’s disease (AD), Parkinson’s disease (PD), and amyotrophic lateral sclerosis (ALS), are characterized by a progressive loss of neuronal structure and function, leading to cognitive and motor impairments [1,2]. AD involves progressive memory loss and cognitive decline. It is marked by the accumulation of amyloid-beta plaques and tau protein tangles, causing synaptic dysfunction and neuronal death [3,4]. PD primarily affects motor control through the degeneration of dopaminergic neurons in the substantia nigra, with alpha-synuclein aggregates (Lewy bodies) playing a key role [5]. ALS primarily affects motor neurons, characterized by the pathological accumulation of TDP-43 protein and genetic mutations that disrupt cellular homeostasis, leading to the gradual loss of motor neurons and ultimately resulting in muscle weakness and paralysis [6].

The prevalence of these neurodegenerative diseases is rapidly increasing worldwide, driven mainly by aging populations. For example, PD affects over 10 million people globally, with incidence expected to double in the next 25 years. This increase is expected to be most pronounced in older age groups, particularly those aged 80 and above. It varies across regions and socioeconomic strata, with middle Socio-demographic Index countries experiencing the highest relative rises [7]. Taken together, these trends underscore a looming global health crisis, with neurodegenerative diseases poised to become an even greater cause of morbidity, disability, and healthcare expenditure in the coming decades. Addressing this challenge will require coordinated research, healthcare infrastructure, and policy efforts to manage the growing patient population effectively.

**Figure 1:**
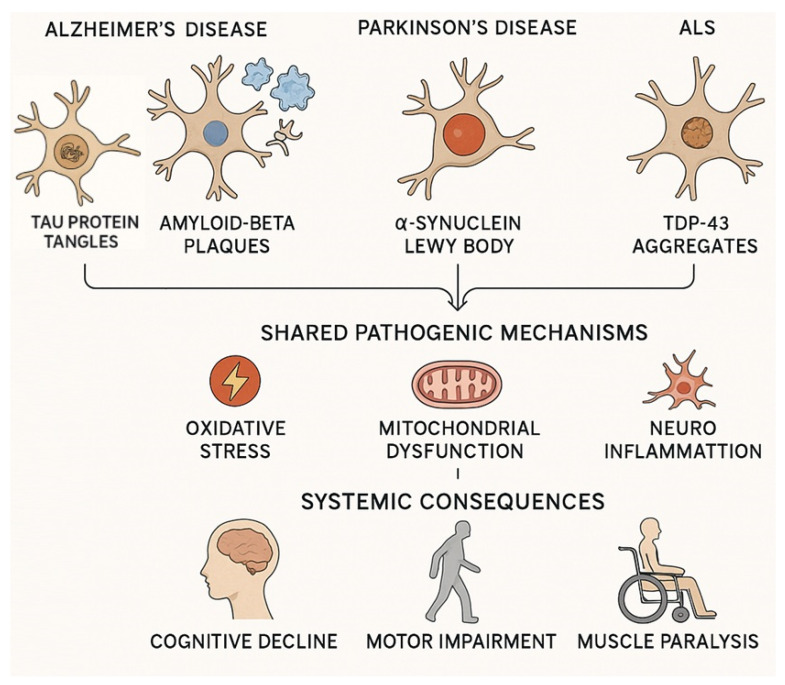
Pathological hallmarks and shared mechanisms in major neurodegenerative diseases. The figure illustrates the key pathological features of Alzheimer’s disease (tau protein tangles and amyloid-beta plaques), Parkinson’s disease (α-synuclein Lewy bodies), and amyotrophic lateral sclerosis (TDP-43 aggregates). Despite distinct protein abnormalities, these disorders share standard pathogenic mechanisms—oxidative stress, mitochondrial dysfunction, and neuroinflammation—which collectively drive systemic consequences such as cognitive decline, motor impairment, and muscle paralysis.

Despite their distinct clinical manifestations, these disorders share standard pathogenic mechanisms (Figure 1). Oxidative stress (OS), mitochondrial dysfunction, and inflammation are central to the pathogenesis [8,9,10]. OS arises from an imbalance between the production of reactive oxygen species (ROS) and reactive nitrogen species (RNS) and the capacity of the cell’s antioxidant defenses to neutralize them. The brain is particularly vulnerable to oxidative damage due to its high oxygen consumption, abundant lipid content, and relatively low antioxidant capacity. Excessive ROS and RNS cause oxidative modification of lipids, proteins, nucleic acids, and other cellular components, leading to cellular dysfunction and death. For example, lipid peroxidation generates toxic by-products such as 4-hydroxy-2,3-nonenal (HNE), which can impair neuronal glucose and glutamate transporters, exacerbating neuronal injury [11,12]. Mitochondrial dysfunction is both a source and a target of OS in neurodegeneration. Impaired mitochondrial respiratory chain function leads to decreased ATP production and increased electron leakage, resulting in the formation of ROS, thereby creating a vicious cycle of oxidative damage. Mitochondrial damage also triggers apoptotic pathways, contributing to neuronal loss. Moreover, mitochondrial dysfunction disrupts calcium homeostasis and impairs energy metabolism, which is critical for neuronal survival and function [13].

Chronic neuroinflammation, driven mainly by activated microglia and astrocytes, amplifies OS and neuronal damage. Activated glial cells release pro-inflammatory cytokines, chemokines, and additional ROS/RNS, perpetuating a harmful inflammatory environment. This neuroinflammatory response disrupts neuronal homeostasis, promotes protein misfolding and aggregation, and impairs the blood–brain barrier, all of which contribute to progressive neurodegeneration [9,12]. OS exacerbates neuronal damage, while chronic neuroinflammation, driven by activated microglia and astrocytes, further promotes neurodegeneration by releasing pro-inflammatory cytokines and disrupting neuronal homeostasis [14]. Together, these interconnected processes contribute to the progressive nature of neurodegenerative diseases and represent key targets for therapeutic intervention.

### 1.2. Ketogenic Metabolism and Neuroprotection

Ketone bodies (KBs) are water-soluble molecules generated by the liver from the breakdown of fatty acids (FAs), especially during limited carbohydrate intake, such as fasting, extended physical activity, or following a ketogenic diet (KD). Once produced, they enter the bloodstream and are delivered to peripheral tissues—including the brain, heart, and muscles—where they are converted into acetyl-CoA to enter the citric acid cycle and support ATP production [15].

KD is a high-fat, low-carbohydrate, and moderate-protein dietary approach that shifts the body’s primary energy source from glucose to fat. This metabolic shift reduces blood glucose and insulin levels, prompting the liver to initiate ketogenesis—the production of KBs such as acetoacetate, acetone, and beta-hydroxybutyrate (BHB). These molecules serve as alternative energy substrates, particularly for the brain, during periods of carbohydrate restriction. This adaptation not only supports energy needs under glucose-limited conditions but also holds therapeutic potential for conditions such as epilepsy, neurodegenerative diseases, and various metabolic disorders [16].

BHB is the most abundant and extensively studied, recognized as an efficient energy substrate and a signaling molecule that influences various metabolic and cellular processes. BHB readily crosses the blood–brain barrier and serves as a crucial fuel for the brain and other tissues during states of carbohydrate scarcity [17]. Beyond its role in energy metabolism, BHB has garnered increasing attention for its potential neuroprotective effects, including modulation of inflammation, reduction in OS, and support of mitochondrial function, making it a central focus in the exploration of ketogenic strategies for the management of neurodegenerative diseases [15].

### 1.3. Aims of the Review

This paper aims to comprehensively summarize the mechanisms of action by which ketogenic metabolism influences neurodegenerative diseases, focusing on the molecular, cellular, and metabolic pathways involved in neuroprotection. It aims to critically evaluate the therapeutic relevance and potential of ketogenic metabolism and ketogenic diets in the context of major neurodegenerative disorders, including AD, PD, and ALS. The paper presents and analyzes current experimental and clinical evidence supporting the use of KBs and ketogenic interventions in improving brain metabolism, reducing OS, modulating inflammation, and enhancing synaptic function in these conditions. Additionally, it discusses and compares various therapeutic approaches, including ketogenic diets and exogenous ketone supplements, highlighting their clinical feasibility, benefits, limitations, and challenges associated with their implementation. The paper also aims to identify existing knowledge gaps and methodological limitations in current research, while proposing future directions for developing personalized ketogenic therapies tailored to individual factors, such as age, sex, disease stage, and genetics. Ultimately, it offers a critical perspective on the translational potential of ketogenic metabolism as a promising strategy for treating neurodegenerative diseases, thereby encouraging further high-quality preclinical and clinical studies.

## 2. Ketone Bodies and Brain Metabolism

### 2.1. Ketogenesis and Ketone Bodies

Ketone bodies are a group of small, water-soluble organic chemical compounds synthesized from fatty acids in the mitochondria of hepatocytes [18,19]. This group is represented by acetone and the 4-carbon carboxylic acids: acetoacetate (AcAc) and BHB [20]. AcAc and BHB play the most significant biological role as alternative energy substrates for extrahepatic tissues. Approximately 70% of the circulating pool of KBs consists of BHB [19].

Under normal conditions, most of the energy requirements of mammals come from carbohydrates. Glucose is used as a substrate for energy production, specifically to generate ATP. This process is crucial for the brain’s proper functioning, which accounts for approximately 20% of the body’s total energy expenditure at rest [21]. In certain situations, such as fasting, starvation, intense exercise, a low-carbohydrate diet, pregnancy, or the neonatal period, the demand for glucose may exceed its supply, and the body must find another source of energy [22]. FAs are mobilized from adipocytes, which are then taken up by the liver, where they undergo beta-oxidation to acetyl-CoA. With high availability of acetyl-CoA and low availability of oxaloacetate, acetyl-CoA is not directed to the tricarboxylic acid (TCA) cycle but to an alternative pathway, ketogenesis. The enzyme acetyl-CoA thiolase enables the condensation of two acetyl-CoA molecules to AcAc. The condensation of acetyl-CoA and AcAc with the participation of 3HMG-CoA synthase produces hydroxymethylglutaryl-CoA (HMG-CoA). HMG-CoA is cleaved into acetoacetate and acetyl-CoA by HMG-CoA lyase. Acetoacetate can be metabolized in two ways: reduced to BHB or a small part spontaneously decarboxylated to acetone [23,24,25]. KBs produced in the liver are released into the circulatory system, from where they are transported to extrahepatic tissues to serve as a source of energy. Acetone can also be excreted through the lungs during respiration, and AcAc is used in the cytoplasm for cholesterol synthesis and lipogenesis [23]. The transport of KBs across the BBB is controlled by monocarboxylic acid transporters—MCT1 and MCT2, which are responsible for regulating the permeability of small monocarboxylic acids. Their expression increases in response to increasing concentrations of ketones in the blood [25,26,27]. This group of compounds not only plays a key role in preventing brain malnutrition in adults but also, together with glucose, serves as a primary energy substrate for the fetal brain, playing a crucial role in brain tissue development [28].

Ketogenesis is the body’s response to altered energy conditions—the body activates regulatory mechanisms that lead to the development of ketogenesis as an alternative energy source. The primary role in the regulation of fatty acid beta-oxidation is played by peroxisome proliferator-activated receptors alpha (PPAR-α) [29]. Its activation by long-chain fatty acids (LCFAs) promotes these processes in mitochondria and peroxisomes. It induces the expression of genes encoding enzymes involved in β-oxidation, such as carnitine palmitoyltransferase (CPT-1). Many cofactors affect PPAR-α activity. Peroxisome proliferator-activated receptor gamma coactivator-1 alpha (PGC-1α) and SIRT, which deacetylate PGC-1, increase its activity and promote PPARα transcriptional activity, thereby supporting beta-oxidation. On the other hand, mTORC1 activation that appears after a meal inhibits PPARα activity, β-oxidation, and ketogenesis [25,30]. Although the synthesis of ketone bodies is a protective mechanism, their excessive production can lead to potentially life-threatening disorders such as diabetic ketoacidosis (DKA), starvation ketoacidosis (SKA), and alcoholic ketoacidosis (AKA) [31].

### 2.2. Energy Utilization in the Brain

KBs delivered to the brain are oxidized in mitochondria by β-hydroxybutyrate dehydrogenase 1 (BDH1) to AcAc, which is then catabolized by succinyl-CoA:3-oxoacid-CoA transferase (SCOT) to AcAc-CoA. The liver is the only organ in the human body where there is no SCOT—it cannot use KBs as an energy source. The next step is a reaction catalyzed by acetoacetyl-CoA thiolase 1 (ACAT1), the products of which are two acetyl-CoA molecules. They convert to citrate and enter the TCA cycle. ATP is produced in the electron transport chain (ETC) [19,21,32]. Cultured rodent brain cells can utilize KBs for oxidative metabolism at a rate much faster than they use glucose. In humans, brain glucose utilization was shown to decrease following KBs administration, while oxygen consumption was the same, suggesting that KBs can be very rapidly substituted for glucose as an alternative energy source for the brain [19,21]. In addition to the leading site of synthesis, KBs can also be synthesized in astrocytes. These cells are the only source of KBs in the brain. Mitochondrial beta-oxidation of FA occurring in astrocytes is a process that protects the brain when glucose is insufficient. The acetyl-CoA produced as a result undergoes ketogenesis to form KBs. After being transported to neighboring neurons, they undergo ketolysis, thereby protecting them in conditions of deficiency [33]. The transport of KBs in brain tissue also occurs via MCTs—MCT4 is expressed in astrocytes, MCT2 in neurons, and MCT11 in brain capillaries [26,34].

### 2.3. Metabolic and Epigenetic Effects

KBs show potential as signaling molecules, influencing mitochondrial function, gene expression, and redox balance, thereby exhibiting neuroprotective, anti-inflammatory, and anti-cancer effects [24,35]. KBs, and especially BHB, exhibit direct and indirect effects as a signaling molecule. Direct effects include inhibition of class I histone deacetylase (HDAC), β-hydroxybutyrylation (BHBylation) of proteins, and effects on cell surface receptors: HCAR2 and FFAR3, membrane channel, and transporter regulation. Indirect effects include the production of acetyl-CoA, GABA, succinyl-CoA consumption, and cytoplasmic NAD^+^ sparing [24].

The HDAC protein family is made up of small, mainly nuclear proteins that are one of the factors modifying gene expression. Their role is to deacetylate lysine residues. BHB acts as a competitive enzyme inhibitor by increasing acetylation, specifically affecting the hyperacetylation of lysines 9 and 14 of histone H3, which weakens DNA–histone connections. As a result, chromatin is loosened, which allows transcription to occur. In a study on mice administered BHB using an osmotic pump, increased histone hyperacetylation in kidney cells and induction of the *Foxo3a* and *MT2* genes were demonstrated; however, further research is needed on this topic. HDAC also deacetylates non-histone proteins such as NF-κB, TP53, MYC, and MYOD1 [24,36,37,38,39].

BHB also affects the functioning of mitochondria, for example, by increasing the activation threshold of the mitochondrial permeability complex (mPT) responsible for cell death. Ketone metabolism helps maintain the correct NAD+/NADH ratio, shifting the balance towards NADH. This enables the activation of sirtrulins, which are regulatory proteins, and supports the pentose phosphate pathway (PPP), responsible for the regeneration of glutathione (GSH)—a key antioxidant. KBs also exhibit direct antioxidant properties as scavengers of free hydroxyl radicals. Reducing their amount can significantly modify signaling pathways and cell damage processes. This effect is supported by the activation of gene expression in the *FOXO3* network, which leads to the induction of antioxidant genes, such as *catalase* and *SOD2*. The interaction of these mechanisms reduces OS [24,40].

Recent preclinical data confirm that BHB can modulate autophagy and mTOR signaling via C99 in an in vivo fruit fly model of AD, suggesting novel mechanisms beyond its classical bioenergetic role [41].

The anti-cancer effect of KBs is also suggested when using a KD because some cancer cells are unable to produce energy from KBs, which, with a limited supply of glucose, may lead to their starvation. KBs also reduce insulin-like growth factor (IGF-1) and insulin, which are essential mitogenic signals, reduce angiogenesis and inflammation, and promote changes in the expression of proteins key to carcinogenesis [42].

The anti-inflammatory potential of KBs has also been demonstrated by inhibiting the NLRP3 inflammasome, reducing the synthesis of IL-1β, IL-2, IL-4, IL-6, TNFα, and NF-κB, and inhibiting the activities of cyclooxygenase-2 (COX-2) [35,43].

## 3. Mechanisms of Neuroprotection

### 3.1. Mitochondrial Enhancement and Oxidative Stress Reduction

Mechanisms of neuroprotection primarily involve enhancing mitochondrial function and reducing OS, both of which are crucial for safeguarding neurons against damage in neurodegenerative diseases and neurological injuries. Combining strategies that strengthen mitochondrial function, through preconditioning and metabolic modulation, with a targeted reduction in OS via antioxidant pathways, offers a promising approach to neuroprotection [44,45]. Mitochondrial antioxidant enzymes like manganese SOD2 neutralize superoxide radicals within mitochondria, while cytosolic and intermembrane space SOD1 also control ROS levels [46,47]. Antioxidants serve a dual role by scavenging ROS and suppressing inflammatory responses in glial cells. Compounds like resveratrol increase the expression of SOD enzymes and inhibit ROS-generating enzymes such as NADPH oxidase [48].

It has been demonstrated that ketogenic metabolism greatly improves mitochondrial function, reduces OS, and holds significant potential to slow the progression of neurodegenerative diseases and enhance neuronal survival. KD increases mitochondrial bioenergetics and efficiency by supplying ketone bodies, such as BHB, as an alternative energy source. By reducing mitochondrial complex I activity, this metabolic change decreases the need for glucose and lowers the production of ROS. Furthermore, it has been demonstrated that BHB increases the production of necessary antioxidant enzymes, such as catalase and SOD, strengthening cellular resistance to oxidative damage [39,49]. Increased ROS levels exacerbate the course of neurodegenerative disorders by causing lipid peroxidation, DNA breakage, and neuronal damage [50]. Ketogenic metabolism mitigates these effects by activating PGC-1α, a master regulator of mitochondrial biogenesis and antioxidant defense [50,51].

The potential of KD as a treatment for neurodegenerative disorders has been highlighted by animal studies that show it increases mitochondrial density and decreases oxidative damage in neural tissues [51]. Ketone substances also directly affect mitochondrial dynamics. Ketogenic therapies have been demonstrated to improve mitochondrial fusion and decrease fragmentation, which are essential for preserving cellular homeostasis and good mitochondrial function [52]. Moreover, their capacity to function as effective energy substrates supports the neuroprotective function of ketone bodies in energy-deficient situations. Ketogenic metabolism, for example, maintains ATP levels and decreases excitotoxic damage in models of traumatic brain injury and stroke, highlighting its potential as a treatment [53].

### 3.2. Anti-Inflammatory and Immunomodulatory Effects

Neuroinflammation is a significant cause of neuronal degeneration in conditions including multiple sclerosis (MS), AD, and PD [54]. The central nervous system’s resident immune cells, microglia and astrocytes, are highly vulnerable to metabolic alterations. Chronic neuroinflammation, driven by dysregulated microglia, astrocytes, and peripheral immune cells, exacerbates neuronal damage by releasing pro-inflammatory cytokines (e.g., IL-1β, TNF-α) and ROS [55,56]. Key pathways, such as nuclear factor-kappa B (NF-κB) and Janus kinase signal transducer and activator of transcription (JAK-STAT) activation, amplify these responses, linking OS and protein aggregation (e.g., amyloid-β, α-synuclein) to sustained inflammation [57]. For instance, α-synuclein in PD activates microglia through Fcγ receptors, triggering NF-κB-driven cytokine production that accelerates the loss of dopaminergic neurons. Immunomodulatory strategies aim to rebalance these responses [58]. Regulatory T cells (Tregs) suppress neurotoxic inflammation by downregulating pro-inflammatory mediators [59]. Microglia, traditionally viewed as destructive, also exhibit neuroprotective roles. Activated microglia engage in synaptic stripping, removing dysfunctional neuronal connections to enhance survival signals and improve neural communication. This dual functionality underscores the importance of targeting microglial activity contextually, suppressing harmful inflammation while preserving their reparative functions [58]. Emerging therapies emphasize multi-modal approaches. Combining anti-inflammatory agents with Tregs-boosting interventions or metabolic modulators addresses the complexity of neuroinflammation.

Reducing NF-κB signaling, a crucial pathway in controlling pro-inflammatory cytokines such as TNF-α and IL-1β, is one of the many ways the ketogenic diet has potent anti-inflammatory effects [60]. Furthermore, BHB directly inhibits the nucleotide-binding domain, leucine-rich-containing family, pyrin domain-containing-3 (NLRP3) inflammasome, a crucial mediator of neuroinflammation linked to neurodegenerative disease [61]. Under ketogenic conditions, microglia transition into the anti-inflammatory phenotype, which is associated with tissue repair and neuroprotection [62]. Additionally, astrocytes alter their metabolic activity, reducing the production of inflammatory mediators and creating a more favorable environment for neuronal survival [63].

It has been demonstrated that ketogenic metabolism reduces neuroinflammation markers, such as glial fibrillary acidic protein (GFAP) and ionized calcium-binding adaptor molecule 1 (Iba1), in preclinical models of neurodegenerative disorders. This lowering correlates with better neuronal integrity and cognitive function [64,65]. Additionally, ketone bodies affect systemic immune responses, which may impact neuroinflammatory diseases such as multiple sclerosis that require peripheral immune responses [66]. New research suggests that ketogenic metabolism influences the gut–brain axis, affecting both central and systemic immunological responses. Reduced neuroinflammation may also result from changes in the gut microbiota composition brought on by KD [67].

Effective neuroprotection requires dampening maladaptive inflammation while harnessing immunomodulatory pathways to support neuronal resilience. Balancing these mechanisms holds promise for slowing neurodegeneration and improving outcomes in chronic neurological disorders.

### 3.3. Effects on Neurotransmission and Synaptic Plasticity

Synaptic plasticity, the dynamic strengthening or weakening of synaptic connections, relies heavily on glutamate receptor activity and calcium signaling [68]. Mechanisms of neuroprotection targeting neurotransmission and synaptic plasticity are essential for preserving cognitive function and neuronal integrity in conditions such as stroke, AD, and traumatic brain injury [69]. In AD, soluble amyloid-β (Aβ) and tau oligomers disrupt synaptic plasticity by interfering with mitochondrial function, vesicle motility, and neurotransmitter release. Therapies targeting these pathologies include compounds that normalize glutamate signaling and approaches to reduce the destabilization of synaptic structures [70].

One of the main consequences of the KD is modification of the equilibrium between excitatory (e.g., glutamate) and inhibitory (e.g., gamma-aminobutyric acid; GABA) neurotransmission. Studies of ketogenic therapies in humans and animals have shown decreased glutamate excitotoxicity and increased GABA levels [71]. This change may help mitigate excitotoxicity in neurodegenerative disorders, reduce neuronal hyperexcitability, and prevent seizure-related damage in epilepsy [72]. Brain-derived neurotrophic factor (BDNF), a crucial modulator of synaptic plasticity and neuronal survival, is also expressed more following a ketogenic diet. Elevated BDNF levels have been associated with improved memory, learning, and resistance to neurodegeneration [73]. Moreover, BHB may help promote synaptic plasticity and upregulate neuroprotective genes through its epigenetic actions, which include inhibiting histone deacetylases (HDACs) [39]. Since synaptic dysfunction occurs before overt neurodegeneration in AD and PD, the ketogenic diet’s capacity to improve synaptic plasticity is especially pertinent in these conditions. Further demonstrating the potential therapeutic benefit of ketogenic treatments, preclinical investigations have shown enhanced memory and cognitive outcomes in models of AD [74,75,76]. Furthermore, ketogenic treatments could promote hippocampal neurogenesis, providing an additional line of defense against neurodegeneration [74,75].

Neuroprotection through neurotransmission and synaptic plasticity involves a multi-faceted approach: balancing excitatory–inhibitory signaling, enhancing energy efficiency via ion channel modulation, leveraging nanotechnology for targeted interventions, and harnessing endogenous neurotrophic and glial support systems. These strategies collectively aim to preserve synaptic integrity and cognitive function in neurological disorders.

**Figure 2:**
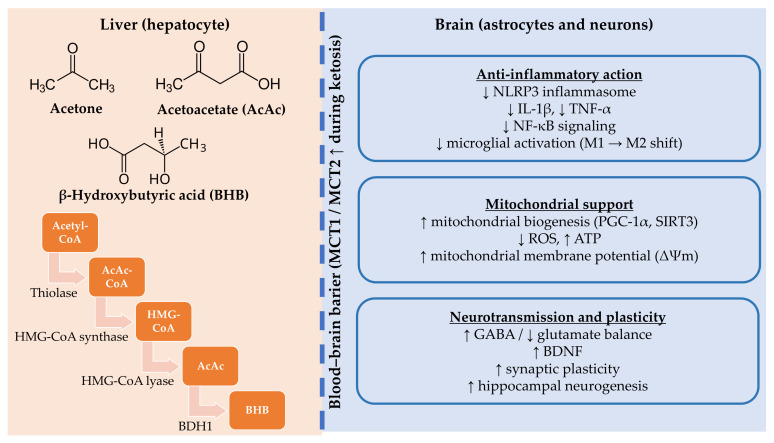
Neuroprotective effects of ketone bodies in neurodegenerative diseases. β-hydroxybutyrate (BHB), produced in the liver from acetyl-CoA via acetoacetate (AcAc) and catalyzed by β-hydroxybutyrate dehydrogenase 1 (BDH1), crosses the blood–brain barrier (BBB) through monocarboxylate transporters (MCT1 and MCT2). In the brain, BHB modulates inflammation by inhibiting NLRP3 inflammasome activation, reducing interleukin-1β (IL-1β), tumor necrosis factor-alpha (TNF-α), and nuclear factor kappa B (NF-κB) signaling, and shifting microglial polarization toward the M2 phenotype. It enhances mitochondrial function by increasing peroxisome proliferator-activated receptor gamma coactivator 1-alpha (PGC-1α) and sirtuin 3 (SIRT3), boosting adenosine triphosphate (ATP) production, reducing reactive oxygen species (ROS), and improving mitochondrial membrane potential (ΔΨm). Additionally, BHB supports neurotransmission and synaptic plasticity by modulating gamma-aminobutyric acid (GABA) and glutamate balance, increasing brain-derived neurotrophic factor (BDNF), enhancing synaptic plasticity, and promoting hippocampal neurogenesis. Arrows indicate the direction of change: ↑ denotes an increase, while ↓ denotes a decrease.

Figure 2 provides an integrated summary of the anti-inflammatory, mitochondrial, and synaptic mechanisms through which ketone bodies may confer neuroprotection in neurodegenerative disorders.

### 3.4. Limitations of Animal Models in Translational Research

While preclinical studies provide critical mechanistic insights into ketogenic metabolism, the extent to which these findings can be extrapolated to human neurodegenerative diseases remains limited. Most rodent models of AD, PD, and ALS reproduce specific pathological features, such as amyloid deposition, tauopathy, α-synuclein aggregation, or motor neuron degeneration, but often fail to fully replicate the progressive, multifactorial, and age-related nature of the human diseases [77,78].

In addition, behavioral endpoints in animals may not accurately reflect human cognitive or motor dysfunction. For example, transgenic models of AD typically demonstrate early amyloid deposition without extensive neurodegeneration, whereas neurotoxin-induced PD models may not reflect the chronic progression observed in humans [79,80,81]. These limitations must be considered when interpreting the efficacy of ketogenic interventions in animal studies and underscore the importance of complementary clinical validation.

## 4. Clinical and Experimental Evidence

### 4.1. Experimental Evidence on Ketogenic Interventions in Alzheimer’s Disease

A defining neuropathological feature of AD is the deposition of extracellular Aβ plaques in cortical and hippocampal regions [82]. The capacity of the KD to influence Aβ accumulation has been explored in several transgenic animal models, yielding divergent results. In one study, short-term implementation of a KD led to a reduction in both Aβ40 and Aβ42 levels in the brains of AD model mice [83]. In contrast, other investigations did not observe a significant decrease in amyloid burden, suggesting that the effectiveness of KD in modulating amyloid pathology may depend on model-specific or methodological variables [84,85,86]. Interestingly, supplementation with exogenous ketone esters in 3xTgAD mice produced a marked decrease in Aβ aggregation, indicating that pharmacological ketosis might exert more substantial anti-amyloid effects than dietary approaches alone.

Evidence regarding the cognitive impact of KD in animal models of AD is similarly mixed. In some models, including APP/V717I, no improvements in memory or learning were reported following KD administration [83]. Furthermore, a study by Park et al. [86] documented a modest deterioration in memory performance in KD-treated animals, which the authors attributed to alterations in gut microbiota composition. In contrast, research involving 3xTgAD mice has demonstrated enhanced cognitive performance following dietary ketosis, suggesting that genetic background and disease stage may modulate the neurocognitive response to ketogenic interventions [87].

Motor function has also been evaluated, albeit less frequently, in AD models. In APP/PS1 knock-in mice, KD improved motor performance but failed to translate into measurable cognitive gains, such as in Barnes maze performance [84]. Comparable findings have been observed in other AD models: both APP/PS1 and Tg4510 mice—representing amyloid and tau pathologies, respectively—exhibited motor improvement in response to KD [85]. In the 5XFAD mouse model, KD has been associated with significant enhancements in spatial learning and memory, effects attributed to improved neuronal integrity within the hippocampus and neocortex [88].

Beyond behavioral outcomes, mechanistic studies implicate KBs—particularly BHB—in modulating key pathological processes in AD [89]. In 5XFAD mice, BHB inhibits activation of the NLRP3 inflammasome, a pro-inflammatory signaling complex that exacerbates Aβ deposition. Exogenous BHB administration reduces plaque burden, attenuates microglial activation, and disrupts inflammasome-related signaling via ASC domain-containing complexes [89]. These findings underscore a potentially disease-modifying role for ketone bodies mediated through anti-inflammatory and immunomodulatory pathways.

Metabolic profiling of AD patients reveals significant alterations, including reduced BHB concentrations in both red blood cells and brain tissue [89]. KD has been shown to restore energetic balance by elevating TCA cycle intermediates, such as citrate, α-ketoglutarate, and amino acids, in the hippocampus of 3xTgAD mice [90]. Furthermore, BHB serves as a more efficient mitochondrial substrate than glucose, particularly under conditions of impaired oxidative phosphorylation typical of AD [91].

Recent studies highlight the gut–brain axis as a potentially critical mediator of KD effects in AD. Alterations in gut microbiota composition, including reductions in microbial diversity and shifts toward Proteobacteria dominance, have been observed in Aβ-infused rodent models [86,92]. Given these associations, the modulation of microbiota by KD may contribute to its neuroprotective effects through systemic and central immune regulation.

### 4.2. Clinical Studies on the Ketogenic Diet in Alzheimer’s Disease

Several clinical studies have investigated the therapeutic potential of ketogenic strategies—primarily medium-chain triglyceride (MCT) supplementation and ketogenic dietary patterns—in patients with AD, particularly in its early stages.

Supplementation with MCTs in combination with leucine and vitamin D has been shown to significantly improve scores on the Mini-Mental State Examination (MMSE) and the Nishimura Geriatric Rating Scale for Mental Status (NM) in frail elderly individuals. Notably, these cognitive benefits appeared to be independent of changes in muscle strength [93]. A subsequent study by the same research group replicated these findings, again reporting significant increases in MMSE and NM scores following MCT administration [94]. These results suggest that MCT-based supplementation may serve as an effective alternative energy source for the brain, particularly in the early stages of AD [95].

AC-1204, a caprylic triglyceride formulation designed to induce mild ketosis, has also been tested in randomized controlled trials. However, no disease-modifying effects were observed in apolipoprotein E4 (*APOE4*)-negative individuals, highlighting the importance of genetic stratification in evaluating therapeutic responses [96]. In another randomized crossover trial comparing a modified KD to a low-fat control diet, participants following the ketogenic regimen experienced improved daily functioning and quality of life, although cognitive performance showed only a non-significant trend toward improvement [97].

The Alzheimer’s Disease Assessment Scale—Cognitive Subscale (ADAS-Cog), widely regarded as the gold standard for quantifying cognitive impairment in AD, has shown responsiveness to ketogenic interventions in multiple studies. KD and MCT-based approaches have been associated with improvements in ADAS-Cog scores, particularly in patients without the *APOE4* allele [98,99]. Moreover, research by Brandt et al. [100], employing a modified Atkins diet, demonstrated selective enhancement of memory performance in early-stage AD patients, though general cognition remained unaffected. Participants also reported improvements in mood and perceived quality of life, underscoring the broader impact of ketogenic interventions beyond cognition alone.

In patients with mild cognitive impairment (MCI), metabolic dysfunction and systemic inflammation are frequently observed comorbidities. Importantly, ketogenic MCT supplementation was found not to adversely affect cardiometabolic or inflammatory markers in this population, suggesting a favorable safety profile [101]. A systematic review (2024) of randomized trials of MCT or exogenous ketones in people with MCI and mild AD showed improved cognitive function. Still, heterogeneity of designs and limited sample sizes do not allow for pooling conclusions [102]. In turn, Shabbir et al. [103] highlighted the neuroprotective mechanisms of ketogenic intervention, including the impact on mitochondrial efficiency and OS, which confirms the growing interdisciplinary perspective of the approach.

Genetic factors, particularly *APOE* genotype, appear to modulate responsiveness to ketogenic therapy. *APOE4* homozygotes may derive greater benefit from KD than non-carriers [104]. Conversely, MCT supplementation has been shown to improve ADAS-Cog scores in *APOE4*-negative individuals, whereas *APOE4*-positive participants did not exhibit the same cognitive gains [99]. Further support for genotype-dependent effects of ketogenic therapy comes from a study on AC-1202, a caprylic triglyceride formulation that induces mild nutritional ketosis. In a multicenter, placebo-controlled trial, Henderson et al. [105] demonstrated that AC-1202 supplementation improved ADAS-Cog scores in patients with mild-to-moderate AD who were *APOE4*-negative, while no benefit was observed in *APOE4*-positive individuals. This underscores the relevance of APOE4 status in determining patient responsiveness to ketogenic agents and highlights the need for personalized intervention strategies.

A comprehensive meta-analysis published in 2024 synthesized data from 10 randomized controlled trials, comprising 691 patients with AD, of whom 651 completed the interventions (357 in KD groups and 334 in control groups) [76]. The analysis demonstrated significant improvements across key cognitive outcomes, including the MMSE, the ADAS-Cog, and the NM, thereby supporting the efficacy of ketogenic interventions in enhancing mental and cognitive function in AD.

Despite promising findings, long-term adherence to the KD presents a significant challenge, particularly in cognitively impaired individuals. Caregivers of patients with moderate-to-advanced AD often report difficulty implementing and maintaining strict dietary regimens. Inconsistent carbohydrate restriction can impair ketosis and diminish the potential therapeutic effects [98].

### 4.3. Experimental Evidence on Ketogenic Interventions in Parkinson’s Disease

One of the proposed mechanisms by which the KD exerts neuroprotective effects in PD involves enhancing mitochondrial metabolism and supporting dopamine biosynthesis. Specifically, KD-induced elevations in circulating KBs stimulate the conversion of tetrahydrobiopterin-3 (BH3) to tetrahydrobiopterin-4 (BH4), thereby increasing the availability of BH4, an essential cofactor for tyrosine hydroxylase (TH)—the rate-limiting enzyme in dopamine synthesis [106]. This mechanism is particularly relevant given that BH4 levels are often reduced in PD patients [107], and their restoration may facilitate dopaminergic neuron activity [108].

In addition to promoting dopamine synthesis, KD has been shown to modulate key intracellular signaling pathways involved in neuronal survival. In animal models of lipopolysaccharide-induced PD, KD activates the AKT/GSK-3β/CREB pathway, resulting in reduced neuroinflammation and attenuation of dopaminergic neuron loss. Notably, this effect appears to be more pronounced when the diet is introduced as a preventive measure rather than as a treatment after symptom onset [109].

Further evidence for the neuroprotective potential of KD comes from studies using the MPTP mouse model of PD. MPTP induces degeneration of dopaminergic neurons in the substantia nigra and mimics Parkinsonian motor deficits. In this model, KD significantly reduced neuronal loss and improved motor function, supporting its role in maintaining nigrostriatal integrity [110].

Induction of ketosis through exogenous supplementation has emerged as an alternative to strict dietary adherence. Compounds such as ketone esters [108] and MCT oil [111] have been used to elevate serum levels of BHB without dietary modification. A ketone ester-enriched diet (KEED) has been shown to delay motor decline in the MitoPark (MP) mouse model of PD, although it does not prevent dopaminergic cell death. KEED also mitigated reductions in dopamine release in these animals [108]. However, some studies suggest that BHB elevation alone may be insufficient to replicate the full neuroprotective effects of KD. In the 6-hydroxydopamine (6-OHDA) rat model of PD, additional metabolic shifts, such as decreased blood glucose and altered insulin sensitivity, appear to be critical for neuroprotection [112].

The gut–brain axis has gained attention in PD pathophysiology, with gut microbiota dysbiosis (GMD) identified in both patients and animal models. Microbial metabolites, particularly short-chain fatty acids (SCFAs), are known to influence central nervous system function via immune and metabolic pathways [113]. Although the direct impact of KD on GMD in PD remains to be fully elucidated, multiple animal studies suggest that ketogenic interventions can restore microbial balance. In a recent study, transplantation of gut microbiota from KD-fed mice to MPTP-induced PD mice led to improved motor outcomes and increased dopaminergic neuron density, indicating a possible microbiota-mediated mechanism of action [114].

### 4.4. Clinical Studies on the Ketogenic Diet in Parkinson’s Disease

Several clinical studies have investigated the efficacy of the KD in managing motor and non-motor symptoms of PD (Table 1). In a randomized controlled trial involving 38 PD patients, participants were assigned to either a KD group or a low-fat diet group. Both dietary interventions led to improvements in symptoms as measured by the International Parkinson and Movement Disorder Society Unified Parkinson’s Disease Rating Scale (MDS-UPDRS). However, the KD group demonstrated significantly greater improvement in non-motor symptoms, specifically in MDS-UPDRS part I. No notable differences were observed in motor symptoms (parts II–IV) between the groups [115].

A separate study randomized 14 participants to receive either a high-carbohydrate or a low-carbohydrate diet. Although no significant differences were observed in motor function, participants in the low-carbohydrate group scored higher on assessments of lexical access and memory, suggesting a potential cognitive benefit of carbohydrate restriction [116].

In a 12-week trial involving 16 PD patients, those who maintained ketosis throughout the intervention exhibited better scores on the Parkinson’s Anxiety Scale (PAS) and MDS-UPDRS part I. However, there were no differences between the ketogenic and control groups in terms of motor function (parts II–IV) or depression symptoms as assessed by the CESD-R-20 scale [117]. A longer, 24-week open-label study involving seven PD patients reported statistically significant reductions in both anxiety and depression, as well as increased social engagement. Notably, the improvements were limited to non-motor domains on the MDS-UPDRS, with motor scores remaining unchanged. These results suggest that KD may preferentially target non-motor symptomatology in PD [118].

In a randomized feasibility study by Choi et al. [111], 16 patients were administered either an MCT-supplemented KD or a standard diet. Although no significant differences were observed in the TUG test or MDS-UPDRS part II, patients in the KD group subjectively reported improved motor function and increased energy levels, implying a beneficial effect on overall well-being.

In addition to general motor and cognitive symptoms, PD is often accompanied by dysarthria—a speech disorder resulting from impaired motor control of the vocal musculature. A study by Koyuncu et al. [119] involving 68 PD patients demonstrated that KD significantly improved voice-related quality of life, as reflected in enhanced scores on the Voice Handicap Index-10 (VHI-10).

### 4.5. Ketogenic Strategies in Amyotrophic Lateral Sclerosis: Preclinical and Clinical Evidence

Amyotrophic lateral sclerosis is a fatal neurodegenerative disorder primarily affecting upper and lower motor neurons. One of its hallmark pathophysiological features is impaired mitochondrial glucose utilization in the spinal cord, which results in cellular energy deficits [120]. This bioenergetic dysfunction may be partially compensated by providing alternative substrates such as ketone bodies, particularly BHB, which bypass impaired glycolytic pathways and support mitochondrial ATP production [121].

Metabolic profiling has revealed altered ketone body dynamics in ALS patients compared to healthy controls. While a negative correlation between body mass index (BMI) and serum ketone levels is observed in healthy individuals, ALS patients display a paradoxical positive correlation, suggesting a disease-specific disruption of energy homeostasis [122].

Preclinical studies provide supportive evidence for the neuroprotective effects of ketogenic strategies in ALS. In a 2008 study, Zhao et al. [123] investigated the impact of a KD on SOD1-G93A transgenic mice, a well-established model of ALS. Although the diet did not halt disease progression, it significantly delayed motor decline, preserved motor performance, inhibited motor neuron degeneration, and enhanced mitochondrial ATP production in spinal neurons. Follow-up research by the same team demonstrated that caprylic triglyceride supplementation—a ketogenic compound metabolized into BHB—promoted mitochondrial oxygen consumption and attenuated both motor dysfunction and neuronal loss in the same model [124].

Additional studies have explored combinatory approaches. Ari et al. [125] reported that KD alone improved motor function in SOD1-G93A mice but did not influence disease progression. However, when combined with the Deanna Protocol (DP)—a multi-nutrient supplement regimen including arginine alpha-ketoglutarate, gamma-aminobutyric acid (GABA), ubiquinol, and MCT oil—survival was extended. Another compound, triheptanoin, a MCT with anaplerotic properties, was demonstrated by Tefera et al. [126] to partially prevent lumbar motor neuron death and delay symptom onset in female SOD1-G93A mice.

Despite encouraging preclinical results, human data remain limited. The only registered clinical trial evaluating KD in ALS patients (NCT01016522) [127] was terminated prematurely due to low recruitment. However, several retrospective and epidemiological studies provide indirect support for the potential utility of ketogenic interventions. Dietary patterns characterized by high-carbohydrate and low-fat intake have been associated with increased ALS risk, suggesting that KD might offer protective effects [128]. An extensive retrospective study in a Chinese ALS cohort reported that KD slowed disease progression and improved motor function as measured by the ALS Functional Rating Scale-Revised (ALSFRS-R) [129].

Coconut oil—rich in MCT—has been shown to elevate serum BHB levels in ALS patients more effectively than in healthy individuals [130]. When added to a Mediterranean-style diet, it significantly increased muscle mass in ALS patients [131]. Although the classification of such dietary interventions as “ketogenic” remains a topic of debate [132].

Emerging research has also focused on sirtuins, particularly SIRT3, which play a regulatory role in mitochondrial integrity and ketone metabolism. SIRT3 has been implicated in preventing neuronal apoptosis and mitochondrial fragmentation in ALS models [133]. Furthermore, it regulates ketone body synthesis by deacetylating mitochondrial 3-hydroxy-3-methylglutaryl-CoA synthase 2 (HMGCS2), a key enzyme in ketogenesis [134].

**Figure 3:**
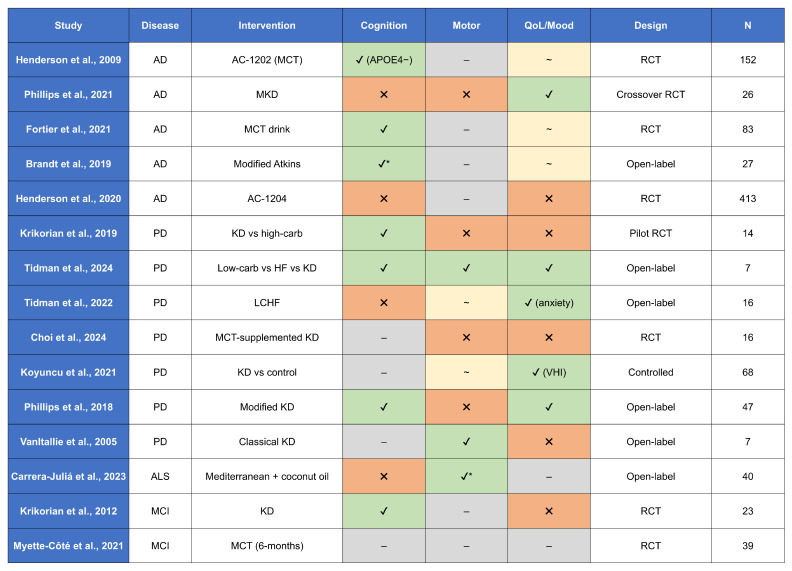
Summary of clinical outcomes in trials of ketogenic interventions across neurodegenerative disorders. Clinical effects are presented by domain: cognition, motor function, and quality of life/mood (QoL). Symbols indicate outcome type: ✔ = significant improvement; ✖ = no effect (assessed); ~ = trend or marginal effect; – = not assessed or not reported. Asterisk (*) indicates that the reported effect was limited to a subset of participants. Abbreviations: AC-1202—medium-chain triglyceride composed of glycerin and caprylic acid; AC-1204—caprylic triglyceride; AD—Alzheimer’s disease; ADL—activities of daily living; ALS—amyotrophic lateral sclerosis; *APOE4*—epsilon 4 variant of the apolipoprotein E gene; KD—ketogenic diet; LCHF—low carbohydrate, high fat diet; MCI—mild cognitive impairment; MCT—ketogenic drink containing medium-chain triglyceride; MKD—modified ketogenic diet; PAS—Parkinson Anxiety Scale; PD—Parkinson’s disease; QoL—quality of life; RCT—randomized controlled trial; VHI—Voice Handicap Index. Data adapted from Henderson et al., 2009 [105]; Henderson et al., 2020 [96]; Phillips et al., 2021 [97]; Brandt et al., 2019 [100]; Fortier et al., 2021 [135]; Krikorian et al., 2019 [116]; Tidman et al., 2024 [118]; Tidman et al., 2022 [117]; Choi et al., 2024 [111]; Koyuncu et al., 2021 [119]; Phillips et al., 2018 [115]; VanItallie et al., 2005 [136]; Carrera-Juliá et al., 2023 [131]; Krikorian et al., 2012 [137]; Myette-Côté et al., 2021 [101].

Table 1 summarizes the key clinical trials evaluating ketogenic interventions in AD. To facilitate interpretation, Figure 3 visually synthesizes the reported clinical effects, study design, and sample size across trials.

**Table 1:** Summary of studies describing the use of the ketogenic diet in patients with neurodegenerative diseases.

Disorders	Study Groups	Intervention	Results	Study
Alzheimer’s disease	152 mild-to-moderate AD patients	oral ketogenic compound AC-1202	improved cognitive outcomes in APOE4 patients; no benefit in APOE4+ carriers	Henderson et al. [105]
Alzheimer’s disease	26 patients with probable AD	modified ketogenic diet vs. usual diet (crossover design)	improved daily function and quality of life; no significant cognitive improvement; mild adverse effects	Phillips et al. [97]
Alzheimer’s disease	83 patients with mild-to-moderate AD	ketogenic MCT drink vs. placebo	significant improvements in verbal fluency, naming, and executive function tests	Fortier et al. [135]
Alzheimer’s disease	MCI or early-stage AD	Modified Atkins Diet	improved episodic memory (Memory Composite Score) in participants achieving ketosis; increased energy levels; feasibility limited by adherence challenges	Brandt et al. [100]
Alzheimer’s disease	413 patients with Mild-to-Moderate AD	AC-1204	no improvement in cognitive and functional abilities in people with mild to moderate AD	Henderson et al. [96]
Parkinson’s disease	14 PD patients	ketogenic diet vs. high-carbohydrate diet	enhanced cognitive performance	Krikorian et al. [116]
Parkinson’s disease	7 PD patients	low-carbohydrate vs. healthy fat vs. ketogenic diet	enhanced cognition, mood, motor and nonmotor symptoms, and reduced pain and anxiety	Tidman et al. [118]
Parkinson’s disease	16 PD patients	LCHF diet	improved scores on the PAS anxiety scale, no improvements on the CESD-R-20 scale for symptoms of depression in the 12 weeks, positive trends for reducing overall PD symptoms, improving biomarkers of chronic disease, and reducing anxiety in persons with PD	Tidman et al. [117]
Parkinson’s disease	16 PD patients	medium-chain triglyceride-supplemented ketogenic diet	no significant improvement in motor and mobility results compared to the standard diet	Choi et al. [111]
Parkinson’s disease	68 PD patients	ketogenic diet	improved VHI parameters	Koyuncu et al. [119]
Parkinson’s disease	47 PD patients	modified ketogenic diet	improved non-motor symptoms (especially urinary problems, pain, fatigue, daytime sleepiness), improved cognitive impairment	Phillips et al. [115]
Parkinson’s disease	7 PD patients	ketogenic diet	improved motor scores	VanItallie et al. [136]
Amyotrophic Lateral Sclerosis	40 ALS patients	Mediterranean diet + coconut oil	benefits at the anthropometric level: increased percentage of muscle mass and decreased percentage of fat mass and abdominal skin folds compared to the control diet	Carrera-Juliá et al. [131]
Mild Cognitive Impairment	23 older adults with MCI	ketogenic diet	improved verbal memory performance	Krikorian et al. [137]
Mild Cognitive Impairment	39 participants with MCI completed	6-month ketogenic medium-chain triglyceride supplement	minimal effects on circulating cardiometabolic and inflammatory markers, aside from a significant increase in plasma IL-8 levels of unclear clinical significance	Myette-Côté et al. [101]

Abbreviations: AC-1202—medium-chain triglyceride composed of glycerin and caprylic acid; AC-1204—caprylic triglyceride; AD—Alzheimer’s disease; *APOE4*—epsilon 4 variant of the apolipoprotein E gene; CESD-R-20—the Centre for Epidemiological Studies Depression Scale; IL-8—interleukin 8; LCHF diet—low-carb, high-fat diet; MCI—Mild Cognitive Impairment; MCT—ketogenic drink containing medium-chain triglyceride; PAS—Parkinson Anxiety Scale; PD—Parkinson’s disease; VHI—Voice Handicap Index.

## 5. Therapeutic Approaches and Translational Potential

### 5.1. Ketogenic Diet vs. Exogenous Ketone Supplements

Exogenous ketone supplementation and the KD are two methods for achieving ketosis, each with unique benefits and drawbacks. The KD promotes metabolic adaptability and long-term ketosis by inducing endogenous ketone body synthesis with a high-fat, low-carb diet. The KD has a long history of therapeutic use, particularly in refractory epilepsy and emerging neuropsychiatric conditions [138]. It promotes a sustained metabolic shift by increasing ketone body production and fatty acid oxidation, thereby supporting mitochondrial function and reducing glucose utilization. This dual substrate availability appears to be more effective in specific contexts, such as myocardial glucose suppression, than exogenous ketone administration alone. The KD’s effects are gradual, requiring days to establish stable ketosis, but it also modulates insulin levels and systemic metabolism, which may contribute to its broader physiological benefits [139].

Exogenous ketone supplements offer a convenient and rapid alternative to induce ketosis, often within 30 min to an hour [140]. Ketone esters are more potent than ketone salts, typically producing higher and longer-lasting ketone levels with fewer side effects like gastrointestinal discomfort or electrolyte imbalances. However, despite their ability to quickly raise circulating ketones, exogenous ketones may not fully replicate the metabolic effects of the KD, such as the promotion of fatty acid oxidation or sustained insulin modulation [141,142]. Some studies suggest that the acute administration of exogenous ketones is less effective in suppressing glucose uptake in tissues such as the heart, potentially due to substrate competition and differences in metabolic signaling [141,142,143].

Both approaches have been explored for various health benefits, including neuroprotection, the management of metabolic diseases, and the enhancement of athletic performance. Clinical research has shown that the KD significantly improves metabolic indices and neurological outcomes; however, long-term adherence is challenging due to its restrictive nature and potential side effects, such as gastrointestinal discomfort and vitamin deficiencies [64]. On the other hand, exogenous ketone supplements rapidly and safely increase blood ketone levels without requiring dietary changes. This method facilitates therapeutic ketosis, especially for patients who are reluctant or unable to follow the KD [144]. However, exogenous supplements have drawbacks, including fluctuating palatability, expense, and the potential for gastrointestinal upset [145]. Exogenous ketones provide a quicker and easier way to reach ketosis. Therefore, they are a supplementary approach rather than a replacement, even if the KD causes more extensive metabolic changes [146].

The KD induces a comprehensive metabolic state through endogenous ketone production and altered fuel utilization, offering sustained and multifaceted benefits. Exogenous ketone supplements provide a rapid and convenient means to elevate ketone levels, but may lack some of the diet’s broader metabolic effects. Choosing between these approaches depends on individual goals, tolerability, and the clinical context, with ongoing research needed to clarify their respective roles and optimize therapeutic strategies.

Ketogenic therapeutic models, including the classical KD, medium-chain triglyceride (MCT)-based diets, and exogenous ketones, differ significantly in ease of use, metabolic effects, and tolerability. KD poses challenges for long-term compliance due to its restrictive nature and complex meal preparation, which often requires professional supervision to avoid nutritional deficiencies [147,148]. In contrast, MCT-based diets offer greater flexibility with higher carbohydrate and protein allowances, thereby improving adherence while maintaining ketosis through the use of MCT oil supplementation [148,149]. Exogenous ketones provide the simplest administration, as they bypass dietary restrictions entirely by directly elevating blood ketone levels via supplements [149].

Achieving ketosis varies across models: CKD typically requires several days to weeks for complete adaptation, whereas MCT-based diets accelerate the onset of ketosis due to the rapid hepatic metabolism of MCTs, often within days [150,151]. Exogenous ketones induce ketonemia most rapidly within hours, although they may suppress endogenous ketone production [152]. Regarding metabolic impacts, both KD and MCT diets reduce fasting glucose and improve insulin sensitivity, as demonstrated in breast cancer and PD [153,154]. However, KD may adversely affect lipid profiles in some individuals, while MCTs show dose-dependent benefits but carry risks of gastrointestinal distress (e.g., diarrhea, abdominal pain) [148,155]. Exogenous ketones have minimal documented effects on glucose or lipids but lack long-term safety data [152].

Side-effect profiles also diverge. KD is associated with long-term risks, such as bone density loss and nutrient deficiencies [155], while MCT-based diets frequently cause transient gastrointestinal symptoms [148]. Exogenous ketones appear well-tolerated in the short term but require further study for chronic use [156]. Crucially, direct comparative studies among these models are scarce, limiting definitive conclusions about optimal efficacy or safety [148,157]. This evidence gap underscores the need for tailored clinical application, considering individual patient factors such as disease context and lifestyle.

### 5.2. Potential for Combined Interventions

The potential for combined ketogenic therapies in neurodegenerative disorders is increasingly recognized as a promising strategy to slow disease progression and improve neurological function. Emerging research is supporting the synergy between additional neuroprotective therapies and ketogenic methods. For example, combining the KD with antioxidants such as N-acetylcysteine or coenzyme Q10 may improve mitochondrial function and lower OS more than each treatment alone does [158,159]. The effects of ketogenic treatments on cellular energy metabolism and repair processes can also be enhanced by the inclusion of neuroprotective drugs, such as nicotinamide adenine dinucleotide (NAD^+^) precursors [160]. Combining ketogenic therapy with traditional treatments in clinical settings has encouraging opportunities. The KD’s potential to improve treatment efficacy is demonstrated by the fact that, in refractory epilepsy, adjunctive administration of the KD in conjunction with anti-seizure drugs has been shown to lower seizure frequency [161]. Additionally, early research suggests potential advantages in neurodegenerative conditions, such as PD and AD, where KD may enhance the effects of dopaminergic or cholinesterase inhibitors [162,163].

Combined ketogenic therapies hold significant potential in managing neurodegenerative disorders by enhancing mitochondrial efficiency, reducing OS and inflammation, and supporting neuronal survival. Continued investigation into their mechanisms and clinical applications will be crucial to fully harnessing their therapeutic benefits. Further study is needed to define interactions and pinpoint the patient subgroups who stand to gain the most from combination protocol optimization. Additionally, integrating ketogenic therapies with other interventions, such as anti-inflammatory agents, antioxidants, or neurotrophic factors, may provide synergistic effects. This multimodal strategy may be more effective in addressing the complex pathophysiology of neurodegeneration than single treatments. Future research should focus on personalized approaches that consider individual metabolic profiles and disease stages to tailor ketogenic interventions for optimal neuroprotection and functional recovery.

### 5.3. Barriers to Implementation

Although ketogenic therapies have therapeutic potential, several obstacles prevent their widespread use. Given that the efficiency of the KD differs based on individual metabolic profiles, illness stage, and genetic predispositions, patient selection remains a crucial concern [164]. Adherence problems undermine the KD’s long-term viability, especially in those that need more prolonged treatment durations, including those with chronic neurological diseases. There is a lack of long-term clinical trials with standardized protocols. Existing studies often have small sample sizes, high dropout rates, and heterogeneity in diet types and monitoring methods, making it difficult to draw definitive conclusions about efficacy and safety in neurodegenerative populations. Continuous medical supervision, including monitoring lipid profiles, nutrient status, and potential side effects, requires resources and expertise that may not be readily available in all clinical settings [165]. Issues of safety and tolerability also present significant challenges. Metabolic and medical contraindications limit the applicability of ketogenic therapies. Dyslipidemia, nephrolithiasis, and decreased bone density are among the possible adverse effects linked to KD that require close observation and customized dietary modifications [166].

Nutritional concerns also pose a significant obstacle. The KD’s restrictive nature often leads to deficiencies in essential vitamins and minerals typically obtained from carbohydrate-rich foods, such as B vitamins, calcium, and potassium. These deficiencies can exacerbate neurological symptoms or overall health, making careful dietary planning and supplementation necessary [167,168]. Exogenous ketone supplements should be used with caution due to gastrointestinal side effects and the absence of long-term safety evidence, particularly in susceptible groups like older people or people with comorbidities [169,170]. Moreover, pragmatic factors, including cost, accessibility, and the requirement for specialized nutritional assistance, restrict the viability of ketogenic therapy in environments with limited resources. To overcome these obstacles, multidisciplinary efforts are needed to create user-friendly formulations, provide precise rules, and increase access to qualified healthcare practitioners [171].

While ketogenic therapies hold promise for neurodegenerative disorders, their implementation is hindered by challenges related to dietary adherence, nutritional deficiencies, medical contraindications, limited clinical evidence, and the need for specialized monitoring. Overcoming these barriers will require personalized treatment plans, patient education, multidisciplinary support, and further rigorous research to optimize safety and therapeutic benefits. Furthermore, the ketogenic diet’s impact on the blood–brain barrier and its long-term effects on brain health remain incompletely understood, raising concerns about unforeseen adverse outcomes. The diet’s influence on comorbidities common in neurodegenerative patients, such as cardiovascular disease or metabolic disorders, also demands careful evaluation.

### 5.4. Interpretation Challenges Due to Protocol Heterogeneity

Clinical trials investigating ketogenic interventions vary considerably in design, dietary composition, duration of intervention, and patient selection. These differences may explain the inconsistencies in cognitive outcomes and tolerability. For example, studies using short-term or modified KDs often show modest or domain-specific effects, whereas longer interventions may be limited by adherence. Furthermore, patients with earlier stages of disease appear to respond more to ketogenic therapy than those with advanced neurodegeneration. This protocol heterogeneity complicates direct comparison and highlights the need for standardized studies.

## 6. Limitations and Future Directions

### 6.1. Evidence Gaps and Methodological Issues

Despite growing interest and promising preclinical data, the clinical evidence supporting ketogenic interventions in neurodegenerative diseases remains limited. Most published studies are small-scale, short-term, or lack rigorous control groups, making it difficult to draw definitive conclusions about efficacy and safety. There is also considerable heterogeneity in study protocols, including differences in the composition and duration of ketogenic diets, use of exogenous ketone supplements, and patient selection criteria. Variability in outcome measures, ranging from cognitive and motor assessments to diverse biomarkers, further complicates cross-study comparisons. Protocol variability across studies remains a barrier to consistent interpretation [16]. New research on cardiometabolic and neurodegenerative interactions underscores the importance of modeling mechanisms that integrate the effects of nutrition, insulin resistance, cardiovascular health, and cognitive function [172]. Additionally, the mechanisms underlying individual responses to ketogenic therapy are not fully understood, and potential placebo effects cannot be excluded in open-label or non-randomized studies. These methodological limitations underscore the need for large, well-designed randomized controlled trials with standardized protocols and robust endpoints.

### 6.2. Personalized Approaches

Emerging evidence suggests that individual factors such as age, sex, disease stage, and genetic background may significantly influence the response to ketogenic therapies. For example, metabolic flexibility, comorbidities, and baseline nutritional status can affect both the tolerability and effectiveness of ketogenic interventions. Specific genetic variants, such as *APOE4* in AD, may modulate the brain’s ability to utilize ketone bodies or respond to dietary interventions [87,105]. Moreover, the progression and heterogeneity of neurodegenerative diseases necessitate personalized approaches, potentially requiring tailored dietary regimens, dosing strategies for exogenous ketones, and careful monitoring of metabolic and clinical parameters. Future research should prioritize the identification of predictive biomarkers and stratification tools to optimize patient selection and maximize therapeutic benefit.

In addition to common neurodegenerative diseases such as AD, PD, and ALS, ketogenic therapies may also have implications for rare disorders and pediatric populations [173]. Classical and modified KD are already established as effective treatments for drug-resistant epilepsy in children, and overlapping mechanisms such as mitochondrial stabilization and glutamate modulation may be beneficial in metabolic or genetic encephalopathies [174,175,176]. Initial reports and case series have examined ketogenic interventions in conditions such as Leigh syndrome, GLUT1 deficiency, and leukodystrophies. Although data remain limited, these indications warrant further investigation, especially in those cases where conventional therapies are lacking [177,178,179,180,181].

### 6.3. Suggested Future Research

To advance the field, future research should focus on several key areas. First, there is a need for large-scale, multicenter clinical trials with standardized ketogenic protocols and long-term follow-up to assess both the efficacy and safety of this approach across diverse patient populations. Studies should incorporate comprehensive biomarker panels, including metabolic, inflammatory, and neuroimaging markers, to elucidate the mechanisms of action and identify responders. Investigations into the optimal timing, duration, and formulation of ketogenic interventions are warranted, as are studies exploring combination therapies with antioxidants, neuroprotective agents, or conventional treatments. Ultimately, the development of non-invasive biomarkers for monitoring ketosis and neuroprotection could facilitate personalized therapy and enhance clinical outcomes. Addressing these research gaps will be essential for translating ketogenic strategies into effective, evidence-based treatments for neurodegenerative diseases.

### 6.4. Safety Considerations and Population-Specific Risk

Ketogenic interventions are generally well tolerated. However, safety concerns should not be underestimated, especially in older adults and those with comorbidities. Reported adverse events include gastrointestinal discomfort, fatigue, dehydration, electrolyte imbalance, and, in some cases, increased LDL cholesterol or dyslipidemia. While clinically significant ketoacidosis is rare outside of type 1 diabetes, cases have been reported in older patients with type 2 diabetes and reduced insulin reserve [182,183,184].

In patients with metabolic syndrome or cardiovascular disease, close monitoring of lipid profiles, renal function, and hydration status is recommended. Elderly patients may also be at greater risk for sarcopenia or micronutrient deficiencies due to reduced dietary diversity [185,186,187]. Therefore, any ketogenic intervention should be individualized, supervised by a physician, and preceded by screening.

## 7. Conclusions

Ketogenic metabolism represents a promising, multifactorial strategy for targeting core pathogenic processes in neurodegenerative diseases. By improving mitochondrial function, reducing OS, modulating neuroinflammatory pathways, and supporting synaptic plasticity, ketone bodies, particularly BHB, exert a wide range of neuroprotective effects. Experimental studies consistently demonstrate benefits across models of AD, PD, and ALS, including improved cognitive and motor outcomes, enhanced neuronal resilience, and reduced pathological burden. Clinical data, although still limited and heterogeneous, suggest that ketogenic interventions may offer therapeutic value, especially in early disease stages and in patients with favorable metabolic or genetic profiles.

Despite these encouraging findings, several barriers hinder the widespread adoption of ketogenic therapies in clinical practice. These include challenges with long-term dietary adherence, potential adverse effects, variability in patient response, and a lack of standardized protocols in clinical trials. Furthermore, much of the mechanistic insight remains derived from animal models, highlighting the need for translational studies and robust, controlled clinical investigations.

Although various ketogenic strategies differ in mechanisms and practical implementation, no consensus has yet emerged on the most effective model, and further comparative clinical trials are needed. Future research should prioritize personalized approaches that consider individual metabolic flexibility, disease progression, and genetic background. Integration of ketogenic strategies with conventional treatments or adjunctive agents may further enhance therapeutic efficacy. With continued interdisciplinary efforts, ketogenic metabolism may emerge as a viable component of precision medicine in the management of neurodegenerative disorders.

## Data Availability

No new data were created or analyzed in this study. Data sharing is not applicable to this article.

## Abbreviations

The following abbreviations are used in this manuscript:

6-OHDA	6-hydroxydopamine
Aβ	amyloid β
AC-1202	medium-chain triglyceride composed of glycerin and caprylic acid
AC-1204	caprylic triglyceride
AcAc	acetoacetate
AD	Alzheimer’s disease
ALS	amyotrophic lateral sclerosis
APOE4	epsilon 4 variant of the apolipoprotein E gene
BBB	blood–brain barrier
BDH1	β-hydroxybutyrate dehydrogenase 1
BH4	tetrahydrobiopterin-4
BHB	β-hydroxybutyrate
BMI	body mass index
CESD-R-20	The Centre for Epidemiological Studies Depression Scale
COX-2	cyclooxygenase-2
DP	the Deanna Protocol
ETC	electron transport chain
FA	fatty acid
GABA	gamma-aminobutyric acid
GFAP	glial fibrillary acidic protein
HMG-CoA	hydroxymethylglutaryl-CoA
HMGCS2	3-hydroxy-3-methylglutaryl-CoA synthase 2
HNE	4-hydroxy-2,3-nonenal
Iba1	ionized calcium-binding adaptor molecule 1
IGF-1	insulin-like growth factor 1
KBs	ketone bodies
KD	ketogenic diet
KEED	ketone ester-enriched diet
LCFA	long-chain fatty acid
LCHF diet	low-carb, high-fat diet
MCI	mild cognitive impairment
MCT	a ketogenic drink containing medium-chain triglycerides
MMKD	modified Mediterranean-ketogenic diet
MDS-UPDRS	Movement Disorder Society Unified Parkinson’s Disease Rating Scale
mPT	mitochondrial permeability complex
MT2	metallothionein 2
NF-κB	nuclear factor kappa-light-chain-enhancer of activated B cells
NM	Nishimura Geriatric Rating Scale for Mental Status
NLRP3	nucleotide-binding domain, leucine-rich repeat, and pyrin domain containing protein 3 (inflammasome)
OAA	oxaloacetate
OS	oxidative stress
PAS	Parkinson Anxiety Scale
PD	Parkinson’s disease
PGC-1α	peroxisome proliferator-activated receptor gamma coactivator 1-alpha
ROS	reactive oxygen species
RNS	reactive nitrogen species
rCBF	regional cerebral blood flow
SCFA	short-chain fatty acid
SCOT	succinyl-CoA:3-oxoacid CoA transferase
SOD2	superoxide dismutase 2
TCA	tricarboxylic acid
TH	tyrosine hydroxylase
TNF-α	tumor necrosis factor alpha
Tregs	regulatory T cells
TUG	Timed Up & Go test
VHI	Voice Handicap Index
